# Is There a Relationship between Shared Decision Making and Breast Cancer Patients’ Trust in Their Medical Oncologists?

**DOI:** 10.1177/0272989X19889905

**Published:** 2019-12-02

**Authors:** Ellen G. Engelhardt, Ellen M. A. Smets, Irini Sorial, Anne M. Stiggelbout, Arwen H. Pieterse, Marij A. Hillen

**Affiliations:** Medical Decision Making, Department of Biomedical Data Sciences, Leiden University Medical Center, Leiden, Zuid-Holland, the Netherlands; Department of Medical Psychology, Amsterdam Public Health Research Institute, Amsterdam University Medical Centers, University of Amsterdam, Amsterdam, Noord-Holland, the Netherlands; Department of Medical Psychology, Amsterdam Public Health Research Institute, Amsterdam University Medical Centers, University of Amsterdam, Amsterdam, Noord-Holland, the Netherlands; Medical Decision Making, Department of Biomedical Data Sciences, Leiden University Medical Center, Leiden, Zuid-Holland, the Netherlands; Medical Decision Making, Department of Biomedical Data Sciences, Leiden University Medical Center, Leiden, Zuid-Holland, the Netherlands; Department of Medical Psychology, Amsterdam Public Health Research Institute, Amsterdam University Medical Centers, University of Amsterdam, Amsterdam, Noord-Holland, the Netherlands

**Keywords:** breast cancer, shared decision making, trust in oncologist

## Abstract

**Background**. Adjuvant systemic treatment for early stage breast cancer significantly reduces the risk of mortality but is associated with side effects, reducing patients’ quality of life. Decisions about adjuvant treatment are preference sensitive and are thus ideally suited to a shared decision making (SDM) approach. Whether and how SDM affects patients’ trust in their oncologist is currently unknown. We investigated the association between patients’ trust in their oncologist and 1) observed level of SDM in the consultation, 2) congruence between patients’ preferred and perceived level of participation, and 3) patient and oncologist characteristics. **Methods**. Decision consultations (*n* = 101) between breast cancer patients and their medical oncologist were audio-recorded and transcribed verbatim. Patients’ trust in their oncologist was measured using the Trust in Oncologist Scale (TiOS). The observed level of SDM was scored using the 12-item Observing Patient Involvement In Decision Making scale (OPTION-12), preferred level of participation with the Control Preferences Scale, and perceived level of participation with an open question in telephonic interviews. **Results**. The average TiOS score was high overall (mean [SD] = 4.1 [.56]; range, 2.6–5.0). Low levels of SDM were observed (mean [SD] = 16 [11.6]; range, 2–56). Neither observed nor perceived level of participation in SDM was associated with trust. Patients’ preferred and perceived role in decision making was incongruent in almost 50% of treatment decisions. Congruence was not related to trust. A larger tumor size (β = 4.5, *P* = 0.03) and the use of a risk prediction model during the consultation (β = 4.1, *P* = 0.04) were associated with stronger trust. **Conclusion**. Patients reported strong trust in their oncologist. While low levels of SDM were observed, SDM was not associated with trust. These findings suggest it may not be necessary to worry about negative consequences for trust of using SDM or risk prediction models in oncological consultations. Considering the increased emphasis on implementing SDM, it is important to further explore how SDM affects trust in clinical practice.

Shared decision making (SDM) is particularly relevant in situations where no single “best option” exists from a medical perspective or where patients’ weighing of the treatment benefits and harms might vary.^1^ SDM is advocated from an ethical perspective as patients have the fundamental right to participate in decisions about their health.^2^ There is weak empirical evidence that SDM positively affects patients’ quality of life^3^ and affective and cognitive outcomes such as satisfaction and decisional regret.^4,5^ Furthermore, SDM may reduce the use of unnecessary medical treatments.^6^

Critics have warned that SDM may also have unwanted negative consequences for patients. Physicians may induce uncertainty by discussing multiple (treatment) options with patients.^7^ As a consequence, patients might become anxious, uncertain, or less satisfied with the consultation.^8^ Moreover, patients might feel pressured to participate in decision making when they do not want to or feel able to do so.

The potential positive and negative consequences of implementing SDM suggest that SDM may have both a positive and a negative effect on the physician-patient relationship. Systematic evidence exploring whether and in which direction SDM affects the physician-patient relationship is limited. This relation is of particular interest, as trust is one of the crucial indicators of the physician-patient relationship.^9^ Trust has been conceptualized as patients’ optimistic acceptance of a vulnerable situation, in which they expect the physician to behave in their best interests.^10^ Trust might be particularly crucial for patients confronted with a potentially life-threatening illness such as cancer. Cancer patients are particularly vulnerable and need to rely strongly on their care providers.^11^ The evidence so far suggests that if cancer patients’ trust in their physician is strong, patients worry less about treatment and are more likely to adhere to treatment advice.^12^

Physicians’ communication has an important impact on patients’ trust.^13–18^ It is therefore likely that the degree to which physicians attempt to involve patients in decision making influences patients’ levels of trust in them. Only 2 studies so far have investigated the relationship between SDM and trust.^19,20^ Both provide preliminary support that physicians’ engagement in SDM might lead to enhanced trust. However, both studies assessed patient-reported SDM rather than observed levels of SDM as determined by an independent rater. As the agreement between self-reported and observed SDM is known to be poor, it is still unknown how trust relates to observed rates of SDM.^21–23^

Therefore, this study aimed to investigate whether SDM, as rated by independent observers or as perceived by patients, is associated with patients’ level of trust in their oncologist. We hypothesized that a higher observed level of SDM is related to stronger trust. In addition, we tested whether congruence between patients’ preferred and perceived degree of participation in decision making predicts patients’ trust. We hypothesized that the greater the congruence between patients’ preferred and perceived level of participation in decision making, the higher their level of trust in the oncologist. Finally, we explored if patient, oncologist, consultation, or hospital characteristics predicted patients’ level of trust in their oncologist.

## Methods

A secondary analysis was performed of data collected for a multicenter observational study among early stage breast cancer patients consulting a medical oncologist about adjuvant systemic treatment (i.e., chemotherapy, endocrine therapy, or both).^24,25^ For women with stage I and II HER2/neu-negative breast cancer, the expected treatment benefits are generally modest, and foregoing treatment is a medically viable option. Chemotherapy is generally only discussed with women eligible for endocrine therapy if they are younger than 36 years at diagnosis, have a HER2/neu-positive tumor, have tumor-positive lymph nodes, or are for some reason unable/unwilling to take endocrine therapy.^26^ The original study aimed to assess information provision during consultations on adjuvant systemic treatment with and without the use of the online prediction model Adjuvant!^27^ All patients had stage I to III breast cancer and were eligible to receive adjuvant systemic treatment with curative intent after surgical removal of the breast tumor. Patients were recruited at 8 university and general teaching hospitals in the Netherlands. The institutional review boards of the participating hospitals approved the study protocol.

### Procedures

After obtaining informed consent, the consultations during which patients and medical oncologists discussed the decision about adjuvant systemic treatment were audiotaped. The audiotapes were transcribed verbatim. Within 1 week of the consultation, patients were interviewed via telephone, and thereafter patients were asked to complete a patient questionnaire. For the current analyses, we randomly selected 101 of the 287 patients for whom an audiotape of the consultation, telephone interview, and patient questionnaire were available. Sample size was determined based on our aim to include minimally 8 cases for each of the 12 factors considered in our regression analysis.

### Measurements

#### Patient and disease characteristics

Patients’ characteristics were collected using the patient questionnaire—specifically, age, education level, parity, and marital status. Treatment and disease characteristics were extracted from patients’ medical charts—specifically, TNM stage of disease, tumor size (small v. intermediate or large), presence of tumor-positive lymph nodes (yes/no), estrogen receptor status, progesterone receptor status, and HER2/neu receptor status. Hospital affiliation (general teaching hospital or university medical center) and oncologists’ gender were recorded at patient inclusion in the study. Use of Adjuvant! (an online survival calculator) during the consultation (yes/no) and treatment options discussed were extracted from the audiotaped consultations.

#### Patients’ trust in the oncologist

In the patient questionnaire, we used the validated 18-item Trust in Oncologist Scale (TiOS)^28,29^ to measure patients’ level of trust in their medical oncologist after the consultation. The TiOS assesses 4 different dimensions of trust: Competence, Fidelity, Honesty, and Caring. Items are answered on a 5-point Likert scale (1 = *completely disagree* to 5 = *completely agree*). An example of an item is “Your oncologist would only think about what is best for you.” Scores are summed and averaged, with higher scores indicating stronger trust (possible range, 1–5). For up to 2 missing values, we used median imputation. Internal consistency was high; Cronbach’s α = 0.9.

#### Patient participation in treatment decision making

We measured the level of SDM from both an independent observer’s and the patient’s perspective. The observed level of SDM was assessed using the validated 12-item Observing Patient Involvement In Decision Making scale (OPTION-12), a discrete measure of the extent to which health care professionals involve patients in medical decisions.^30^ Items are scored on a 5-point Likert scale (0 = *behavior is not observed* to 4 = *behavior is observed and executed to a high standard*). Sum scores were converted to a 0 to 100 scale in this study. In the present study, internal consistency was sufficient (Cronbach’s α = .8). Transcriptions of consultations were double-coded by 2 researchers until adequate interrater reliability was achieved for all items (Cohen’s κ≥0.7). Analyzing the content of the consultations using this tool is very labor-intensive. Due to time constraints, we opted to select a random sample of 100 patients meeting the 3 selection criteria for these secondary analyses (i.e., for whom an audiotape of the consultation, telephone interview, and patient questionnaire were available). We inadvertently coded 1 consultation more than intended, resulting in a total of 101 consultations used for our analyses.

We assessed the patient’s preferred level of participation in decision making using the Control Preferences Scale (CPS).^31^ Patients were asked in the questionnaire to choose 1 of 5 levels of participation; for the analysis, we grouped the categories into 3 categories as described by Degner et al.^31^: 1) patient driven, 2) shared, or 3) oncologist driven.

Patients’ perceived level of participation in decision making was assessed in the telephone interview using an open-ended question: “Who made the final decision on whether to start treatment X in your opinion?” for chemotherapy and endocrine treatment separately. Patients’ answers were classified into 3 response categories: 1) patient driven, 2) shared, or 3) oncologist driven. The answers were independently categorized by 2 researchers until sufficient interrater reliability was achieved (Cohen’s κ≥0.7). After that, 1 researcher categorized the remaining answers.

### Statistical Analyses

Statistical analyses were performed using SPSS version 25 (SPSS, Inc., an IBM Company, Chicago, IL). For descriptive analyses, means with standard deviations (SDs) were calculated for continuous variables and absolute numbers with percentages for dichotomous variables. For all analyses, a 2-sided *P* value ≤0.05 was considered significant.

A 1-way analysis of variance (ANOVA) was used to test the association between patients’ perceived level of participation in decision making and trust in their oncologist. Pearson’s correlations were calculated to test associations between the observed level of SDM and trust. If an association was found, we planned to perform a multivariate linear regression analysis to quantify the strength of the relationship between SDM and patients’ trust, correcting for patients’ age, oncologists’ level of experience, and disease stage.

Preferred and perceived levels of participation were categorized using the same categories: 1) patient driven, 2) shared, or 3) oncologist driven. We compared these 2 variables to determine whether the preferred and perceived level of participation categories matched or not (categorical variable: congruent v. incongruent, more involvement than preferred v. incongruent, less involvement than preferred). We used Kruskal-Wallis tests to investigate whether congruence between patients’ preferred and perceived level of involvement in medical decisions was associated with patients’ level of trust.

Using linear regression analysis with backward selection, we evaluated which patient sociodemographic and disease characteristics, as well as oncologist, consultation, and hospital characteristics, predicted patients’ level of trust in their oncologist. The variables included in the regression analyses were selected a priori based on expert knowledge and literature. Box 1 provides an overview of the variables considered.

**Box 1:** Variables Included in the Multivariate Linear Regression Analysis

Patient Characteristics	
Age	In years
Education	Low educational level (i.e., none/primary/low-level vocational education)
	Intermediate educational level (i.e., secondary school/intermediate vocational education)
	High educational level (i.e., higher vocational education/university)
Children	Has children
	Has no children
Marital status	Single/divorced/widowed
	In a relationship
Preferred level of involvement in decision making	Patient driven
	Shared
	Oncologist driven
Hospital, Oncologist, and Consultation Characteristics	
Hospital affiliation	General teaching hospital
	University medical center
Oncologist gender	Male
	Female
Use of online survival indicator software during the consultation	Adjuvant! not used
	Adjuvant! used
Consultation duration	In hours and minutes
Disease Characteristics	
TNM stage of disease^a^	Stage I
	Stages II and III
Size of tumor	Tumor 0–20 mm
	Tumor >21 mm
Tumor-positive lymph nodes	Absent
	Present

a TNM staging source: American Joint Committee on Cancer. *Cancer Staging Manual*. 7th ed. Chicago, IL: American College of Surgeons, 2015.

## Results

A total of 101 consultations conducted by 18 oncologists were included, of which 94 (93%) were conducted by a specialist and 7 (7%) by a resident (Table 1). Patients were on average 60 years at diagnosis (SD = 11).

**Table 1:** Sample Characteristics (N = 101)

Characteristic	Value
Patient age in years, mean (SD)	60.6 (11)
TiOS score, mean (SD)	4.1 (0.6)
Consultation duration, mean (SD), min	00:28:03 (00:12:29)
OPTION-12 sum score, mean (SD)	15.5 (11.6)
Education, *n* (%)	
High	29 (29)
Intermediate	51 (50)
Low	21 (21)
Marital status, *n* (%)	
Single	35 (35)
In a relationship	66 (65)
Has children, *n* (%)	84 (83)
Preferred level of involvement in decision making, *n* (%)	
Patient driven	39 (39)
Shared	41 (41)
Oncologist driven	21 (21)
Consultations with university hospital oncologists, *n* (%)	20 (20)
Consultations with use of Adjuvant! online, *n* (%)	60 (59)
Tumor size, *n* (%)	
0–20 mm	58 (58)
≥21 mm	42 (42)
Missing	1
Tumor stage,^a^*n* (%)	
Stage I	43 (43)
Stages II and III	58 (57)
Tumor positive lymph nodes present, *n* (%)	33 (33)
Positive estrogen receptor (ER) status (*n* = 96), *n* (%)	84 (88)
Missing	5
Positive progesterone receptor (PR) status (*n* = 95), *n* (%)	73 (77)
Missing	6
Positive HER2/neu receptor status, *n* (%)	7 (7)
Missing	5
Triple negative (i.e., ER/PR/HER2/neu negative; *n* = 96), *n* (%)	10 (10)
Missing	5
Surgery (n = 96), *n* (%)	
Breast conserving	51 (53)
Mastectomy	45 (47)
Missing	5
Has received radiotherapy (*n* = 96), *n* (%)	54 (56)
Missing	5
Treatment(s) discussed during the consultation, *n* (%)	
Chemotherapy only	12 (12)
Endocrine therapy only	18 (18)
Chemotherapy and endocrine therapy	70 (70)
Chemotherapy decision (yes; *n* = 94), *n* (%)	53 (57)
Missing	7
Endocrine therapy decision (yes; *n* = 93), *n* (%)	79 (85)
Missing	8

OPTION-12, 12-item Observing Patient Involvement In Decision Making scale; TiOS, Trust in Oncologist Scale.

a TNM staging source: American Joint Committee on Cancer. *Cancer Staging Manual*. 7th ed. Chicago, IL: American College of Surgeons, 2015.

### Research question 1: Association between SDM and Trust

**Figure 1:**
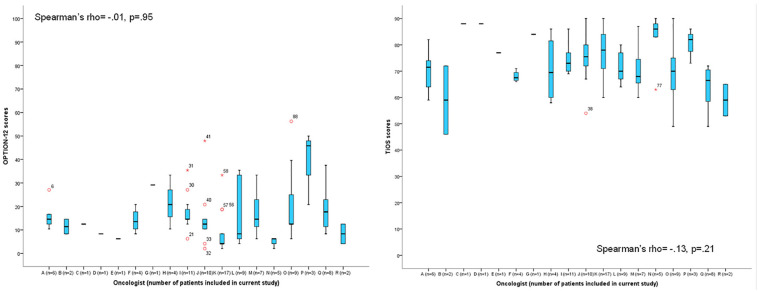
Overview of the distribution of scores on the Trust in Oncologist Scale (TiOS) and the 12-item Observing Patient Involvement In Decision Making scale (OPTION-12) per oncologist.

The mean (SD) TiOS score was 4.1 (.6; range, 2.6–5.0) out of a maximum of 5. OPTION-12 scores were low with a mean (SD) of 15.5 (11.6; range, 2–56) out of a possible 100. Two consultations received an OPTION-12 score ≥50. Given the limited size of our sample, we were unable to account for the hierarchical structure of our data (patients clustered within physicians) by means of multilevel analyses. However, to investigate interdependencies, we plotted the range of both trust (TiOS) and observed SDM scores (OPTION-12) per oncologist (Figure 1). As we observed no trends within clusters that would affect our findings, we continued our analyses without accounting for the hierarchical structure of the data.

Observed level of SDM (OPTION-12) was not correlated with patients’ level of trust in their oncologist (TiOS; Pearson’s *R* = .02, *P* = 0.8), nor was patients’ perception of their participation in decision making (chemotherapy: *F*(1) = 0.1, *P* = 0.8; endocrine therapy: *F*(1) = 0.3, *P* = 0.6). As there was no association between SDM and trust in the oncologist, we did not perform the planned linear regression analyses.

### Research question 2: Association between the Level of Trust and Congruence between Preferred and Perceived Role in Decision Making

About three-quarters of patients preferred some level of control in treatment decision making (Tables 2 and 3). Of the patients eligible for chemotherapy, 66% felt they had made the final decision, compared to 46% of the patients eligible for endocrine therapy. For both chemotherapy and endocrine therapy, 9% of patients explicitly reported having perceived that a shared decision had been made.

**Table 2:** Congruence between Preferred and Perceived Role in Chemotherapy Decision Making

	Perceived Level of Participation, *n* (%)			
Preferred Level of Participation	Oncologist Driven	Shared	Patient Driven	Total
Oncologist driven	7 (47)	1 (7)	7 (47)	15 (21)
Shared	8 (28)	5 (17)	16 (55)	29 (41)
Patient driven	3 (11)	0	24 (89)	27 (38)
Total	18 (25)	6 (8)	47 (66)	71

**Table 3:** Congruence between Preferred and Perceived Role in Endocrine Therapy Decision Making

	Perceived Level of Participation, *n* (%)			
Preferred Level of Participation	Oncologist Driven	Shared	Patient Driven	Total
Oncologist driven	13 (68)	1 (5)	5 (26)	19 (24)
Shared	13 (43)	5 (17)	12 (40)	30 (38)
Patient driven	9 (31)	1 (3)	19 (66)	29 (37)
Total	35 (45)	7 (9)	36 (46)	78

**Figure 2:**
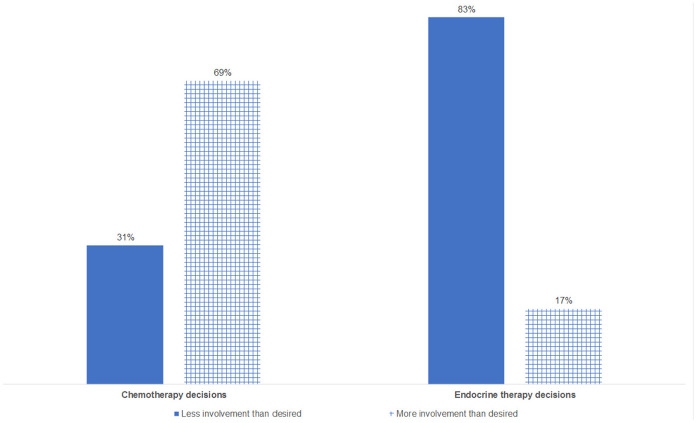
Direction of incongruence between preferred and perceived involvement in decision making. Percentage in bar charts based on consultations in which preferred and observed involvement in decision making were incongruent: chemotherapy (*n* = 35 consultations) and endocrine therapy (*n* = 41 consultations).

Preferred and perceived role in decision making was the same for 51% of chemotherapy and 47% of endocrine therapy decisions. If patients’ preferred and perceived participation were incongruent, then we observed that for 69% of such chemotherapy decisions, patients experienced *more* involvement than preferred, and for 83% of such endocrine therapy consultations, patients experienced *less* involvement than preferred (Figure 2). Level of trust in the oncologist was not associated with congruence between preferred and perceived participation in decision making (chemotherapy: Kruskal-Wallis *H* = 0.7, *P* = 0.7; endocrine therapy: Kruskal-Wallis *H* = 0.7, *P* = 0.7).

### Research question 3: Potential Predictors of Trust in the Oncologist

Table 4 shows the predictive effects of patient sociodemographic and disease characteristics, as well as oncologist, consultation, and hospital characteristics for patients’ level of trust in their oncologist. None of the sociodemographic variables were associated with trust. Larger tumor size (β = 4.5, *P* = 0.03) and the oncologist’s use of Adjuvant!, an online survival calculator (β = 4.1, *P* = 0.04), predicted higher trust in the oncologist.

**Table 4:** Factors Associated with Patients’ Trust in Their Oncologist in Multivariate Linear Regression

Characteristic	B	Standard Error	*P* Value	95% Confidence Interval	
				Lower Bound	Upper Bound
Full model					
(Constant)	72.4	5.6	—	61.2	83.5
Children (no children [reference])	−2.8	2.9	0.3	−8.5	2.9
Marital status (single [reference])	−1.2	2.2	0.6	−5.7	3.2
Preferred level of involvement in decision making					
Patient driven (reference)					
Shared	<0.1	<0.1	1.0	<0.1	<0.1
Oncologist driven	<0.1	<0.1	0.6	<0.1	<0.1
Type of hospital (nonacademic [reference])	−0.8	2.7	0.8	−6.2	4.6
Gender oncologist (female [reference])	−1.9	2.2	0.4	−6.3	2.5
Use of Adjuvant! online (not used [reference])	4.5	2.2	<0.05	0.1	8.8
Duration of consultation (continuous)	<0.1	<0.1	0.3	<0.1	<0.1
Stage (stage 1 [reference])	−5.8	4.7	0.2	−15.1	3.6
Tumor size (smaller than 20 mm [reference])	8.0	3.8	<0.05	0.5	15.5
Nodal status (node negative [reference])	3.1	3.3	0.3	−3.5	9.8
Final model^a^ containing only factors with *P* < .05					
(Constant)	68.6	1.8	—	65.1	72.0
Use of Adjuvant! online (not used [reference])	4.1	2.0	<0.05	0.2	8.1
Tumor size (smaller than 20 mm [reference])	4.5	2.0	<0.05	0.6	8.4

a. The final multivariate regression model was based on stepwise regression with backward selection.

## Discussion

We investigated the relationship between the level of SDM about adjuvant treatment for breast cancer and patients’ trust in their oncologist. Overall, about half of all patients felt they had decided on adjuvant treatment themselves, whereas only a few reported having experienced *shared* decision making. Neither patients’ self-perceived nor observed SDM were related to patients’ trust in their oncologist. Congruence between patients’ preferred and perceived participation in decision making did not predict patients’ trust in their oncologist. Trust levels were higher for patients with larger tumor size and for patients in whose consultations the oncologist had used an online survival calculator.

We did not find an association between SDM and trust. This null finding could be explained by the fact that patients’ trust in their oncologist was high overall, with clear ceiling effects, and the OPTION-12 scores were consistently low, with comparable floor effects. Possibly, in a sample with more variable trust and/or SDM scores, a positive or negative association could be detected. Our key findings are remarkable—namely, that 1) trust levels were high even though adequate levels of SDM were hardly observed, and 2) a significant proportion of patients reported to have made the final treatment decision themselves. It might suggest that patients felt sufficiently comfortable to let the oncologist take the lead while still experiencing a sense of autonomy.^32^ Alternatively, oncologists may have incorrectly interpreted SDM as a full delegation of decisional responsibility to the patient instead of a shared process.^33^ Importantly, the OPTION-12 rates physicians’ efforts to involve patients in decision making, irrespective of the extent to which patients themselves contribute to the decision. Another plausible explanation for this apparent discrepancy is that patients may not have been aware that more than 1 treatment option existed.^34^ Hence, patients in our study may have felt they were making a final decision, even if they were not made aware of other options than the oncologist’s treatment plan. Possibly, an association between SDM and trust may be revealed in samples with more variation in trust or SDM levels. For example, physicians may increase patients’ awareness of uncertainty when informing patients more fully about the pros and cons of multiple possible treatment options. This may, in turn, affect patients’ trust. In addition, results might differ if replicated in other cultures differing in their approach to authority.^35^ For example, patients in the United States may have higher expectations of their involvement in decision making, leading to reduced trust if this expectation is not met.

Patients in our study reported stronger trust if oncologists used the online survival calculator Adjuvant! during the consultation. The use of Adjuvant! may have helped oncologists to better explain the clinical reasoning behind their treatment recommendation to patients and therefore made them seem more trustworthy to patients. Physicians have voiced concern that the prognostic information from survival calculators is too complex or threatening to share with patients. Our findings suggest that such worries may be unnecessary. Presenting complex models and statistics through Adjuvant! may instead enhance patients’ perception of the oncologists’ medical competence. It may moreover strengthen patients’ impression that the oncologist does not convey a personal preference but that the treatment recommendations are based on scientific knowledge and are likely shared by the professional community. Use of Adjuvant! and other similar tools (e.g., PREDICT) may also strengthen patients’ impression that oncologists provided honest and complete information. In line with these hypotheses, both perceived competence and perceived honesty have been previously found to contribute to patients’ trust in an oncologist.^13^ Further research should substantiate the relationship between physicians’ use of risk prediction models and trust and examine how this relationship can be explained. The positive relation between trust and tumor size in this study could be accounted for by vulnerability: patients who had a larger tumor may have felt a stronger need to trust their oncologist^11^ because they felt more vulnerable and dependent. More substantial research is needed to further establish the relationship between trust and disease severity.

The degree of SDM in the present study was consistently low, and it was even lower than levels found in a review of 29 previous studies applying the OPTION-12^36^ and in a previous study assessing SDM among breast cancer patients.^8^ Oncologists in our study may not have perceived their consultations as involving a preference-sensitive decision that requires patient involvement, although most of them should be considered as such: in the Netherlands, a minimum benefit of 4% to 5% absolute gain on survival is the threshold for eligibility for adjuvant therapy.^26^ Roughly 59% of the patients included in our study fall in the category of patients for whom, based on their tumor characteristics, the expected treatment benefit is modest (on average less than 10% absolute benefit). Because of the preventive nature, modest absolute expected benefit, and significant side effects of these adjuvant treatments, these treatment decisions qualify as preference sensitive.^26,37^

## Limitations of Our Study

An important limitation of this study was that our measure of observed SDM, the OPTION-12, only rates the physician’s actions to involve patients in the decision making process. Possibly, also taking patients’ actions and reactions into account would yield more variation in observed levels of SDM. Many other instruments, some of them observation based, are available to assess SDM, but instruments assessing dyadic processes are still lacking.^38^ A second limitation is that we assessed patients’ preferred role in SDM after the consultation. Potentially, patients’ preferences would have differed if they had been asked beforehand, due to post hoc justification: they may have adapted their reported preferences to how the consultation turned out. However, the significant proportion of incongruence between patients’ preferred and perceived role in decision making suggests that post hoc justification does not fully account for patients’ self-reported preference in participation.

Finally, in our data, the patient consultations are clustered within medical oncologists. This could bias our results, as within clusters, the consultations are likely to be more alike than between clusters. However, between medical oncologists, no clear patterns were observed between clusters for the TiOS or OPTION-12 scores. We, therefore, expect that the effect of clustering on our findings is not noteworthy.

## Conclusion

We found high levels of patient-reported trust in their oncologist along with an observed lack of SDM, even in situations where patients preferred higher or lower involvement in decision making than they experienced. This suggests that a lack of patient involvement does not negatively affect the oncologist-patient relationship. However, our findings do not offer definitive indications regarding this relationship. Given the numerous efforts to implement SDM in clinical practice, it is reasonable to expect that physicians will increasingly aim to achieve SDM with their patients. It is therefore imperative to further explore how applying SDM in clinical practice may affect trust and which factors are relevant moderators in this relationship.

## References

[1] Stiggelbout AM Van der Weijden T De Wit MP, et al Shared decision making: really putting patients at the centre of healthcare. BMJ. 2012;344:e256. 22286508 10.1136/bmj.e256

[2] Stiggelbout AM Pieterse A De Haes J Shared decision making: concepts, evidence, and practice. Patient Educ Couns. 2015;98:1172–9. 10.1016/j.pec.2015.06.022 26215573

[3] Kashaf MS McGill E Does shared decision making in cancer treatment improve quality of life? A systematic literature review. Med Decis Making. 2015;35:1037–48. 10.1177/0272989X15598529 26246515

[4] Shay LA Lafata JE Where is the evidence? A systematic review of shared decision making and patient outcomes. Med Decis Making. 2015;35:114–31. 10.1177/0272989X14551638 PMC4270851 25351843

[5] Légaré F Adekpedjou R Stacey D, et al Interventions for increasing the use of shared decision making by healthcare professionals. Cochrane Database Syst Rev. 2018;7:CD006732. 10.1002/14651858.CD006732.pub4 PMC6513543 30025154

[6] Oshima Lee E Emanuel EJ Shared decision making to improve care and reduce costs. N Engl J Med. 2013;368:6–8. 23281971 10.1056/NEJMp1209500

[7] Simpkin AL Schwartzstein RM Tolerating uncertainty—the next medical revolution? N Engl J Med. 2016;375:1713–5. 10.1056/NEJMp1606402 27806221

[8] Politi MC Clark MA Ombao H Dizon D Elwyn G Communicating uncertainty can lead to less decision satisfaction: a necessary cost of involving patients in shared decision making? Health Expect. 2011;14:84–91. 20860780 10.1111/j.1369-7625.2010.00626.x PMC3010418

[9] Emanuel EJ Dubler NN Preserving the physician-patient relationship in the era of managed care. JAMA. 1995;273:323–9. 7815662

[10] Hall MA Dugan E Zheng BY Mishra AK Trust in physicians and medical institutions: what is it, can it be measured, and does it matter? Milbank Q. 2001;79:613–39. 10.1111/1468-0009.00223 PMC2751209 11789119

[11] Hillen MA Onderwater AT Van Zwieten MCB de Haes JC Smets EM Disentangling cancer patients’ trust in their oncologist: a qualitative study. Psychooncology. 2012;21:392–9. 10.1002/pon.1910 21280138

[12] Hillen MA de Haes HC Smets EM Cancer patients’ trust in their physician—a review. Psychooncology. 2011;20:227–41. 10.1002/pon.1745 20878840

[13] Hillen MA De Haes HCJM Stalpers LJA, et al How can communication by oncologists enhance patients’ trust? An experimental study. Ann Oncol. 2014;25:896–901. 24615411 10.1093/annonc/mdu027

[14] Hillen MA de Haes JCJM van Tienhoven G, et al All eyes on the patient—the influence of oncologists’ nonverbal communication on breast cancer patients’ trust. Breast Cancer Res Tr. 2015;153:161–71. 10.1007/s10549-015-3486-0 PMC4536267 26227472

[15] Mechanic D Meyer S Concepts of trust among patients with serious illness. Soc Sci Med. 2000;51:657–68. 10.1016/s0277-9536(00)00014-9 10975226

[16] Wright EB Holcombe C Salmon P Doctors communication of trust, care, and respect in breast cancer: qualitative study. BMJ. 2004;328:864–7. 10.1136/bmj.38046.771308.7C PMC387476 15054034

[17] Butow PN Dowsett S Hagerty R Tattersall MH Communicating prognosis to patients with metastatic disease: what do they really want to know? Support Care Cancer. 2002;10:161–8. 10.1007/s005200100290 11862506

[18] Torke AM Corbie-Smith GM Branch WT Jr. African American patients’ perspectives on medical decision making. Arch Intern Med. 2004;164:525–30. 10.1001/archinte.164.5.525 15006829

[19] Ommen O Thuem S Pfaff H Janssen C The relationship between social support, shared decision-making and patient’s trust in doctors: a cross-sectional survey of 2,197 inpatients using the Cologne Patient Questionnaire. Int J Public Health. 2011;56:319–27. 10.1007/s00038-010-0212-x 21076932

[20] Thüm S Janssen C Pfaff H Lefering R Neugebauer EA Ommen O The association between psychosocial care by physicians and patients’ trust: a retrospective analysis of severely injured patients in surgical intensive care units. Psychosoc Med. 2012;9:Doc04. 10.3205/psm000082 PMC3461762 23049644

[21] Kriston L Härter M Scholl I A latent variable framework for modeling dyadic measures in research on shared decision-making. Z Evid Fortbild Qual Gesundheitswes. 2012;106:253–63. 10.1016/j.zefq.2012.03.021 22749072

[22] Scholl I Kriston L Dirmaier J Härter M Comparing the nine-item Shared Decision-Making Questionnaire to the OPTION Scale—an attempt to establish convergent validity. Health Expect. 2015;18:137–50. 10.1111/hex.12022 PMC5060753 23176071

[23] Kasper J Heesen C Köpke S Fulcher G Geiger F Patients’ and observers’ perceptions of involvement differ: validation study on inter-relating measures for shared decision making. PLoS One. 2011;6:e26255. 10.1371/journal.pone.0026255 PMC3197148 22043310

[24] Engelhardt EG Pieterse AH Han PK, et al Disclosing the uncertainty associated with prognostic estimates in breast cancer: current practices and patients’ perceptions of uncertainty. Med Decis Making. 2017;37:179–92. 10.1177/0272989X16670639 27681991

[25] Engelhardt EG Pieterse AH van der Hout A, et al Use of implicit persuasion in decision making about adjuvant cancer treatment: a potential barrier to shared decision making. Eur J Cancer. 2016;66:55–66. 27525573 10.1016/j.ejca.2016.07.011

[26] Nationaal Borstkanker Overleg Nederland (NABON). Breast cancer guideline. 2017 Available from: https://www.oncoline.nl/borstkanker.

[27] Ravdin PM Siminoff LA Davis GJ, et al Computer program to assist in making decisions about adjuvant therapy for women with early breast cancer. J Clin Oncol. 2001;19:980–91. 10.1200/JCO.2001.19.4.980 11181660

[28] Hillen MA Butow PN Tattersall MHN, et al Validation of the English version of the Trust in Oncologist Scale (TiOS). Patient Educ Couns. 2013;91:25–8. 10.1016/j.pec.2012.11.004 23219483

[29] Hillen MA Koning CCE Wilmink JW, et al Assessing cancer patients’ trust in their oncologist: development and validation of the Trust in Oncologist Scale (TiOS). Support Care Cancer. 2012;20:1787–95. 10.1007/s00520-011-1276-8 PMC3390706 21947560

[30] Elwyn G Hutchings H Edwards A, et al The OPTION scale: measuring the extent that clinicians involve patients in decision-making tasks. Health Expect. 2005;8:34–42. 15713169 10.1111/j.1369-7625.2004.00311.x PMC5060272

[31] Degner LF Kristjanson LJ Bowman D, et al Information needs and decisional preferences in women with breast cancer. JAMA. 1997;277:1485–92. 9145723

[32] McKinstry B Ashcroft RE Car J Freeman GK Sheikh A Interventions for improving patients’ trust in doctors and groups of doctors. Cochrane Database Syst Rev. 2006;3:CD004134. 10.1002/14651858.CD004134.pub2 16856033

[33] Adsul P Wray R Boyd D Weaver N Siddiqui S Perceptions of urologists about the conversational elements leading to treatment decision-making among newly diagnosed prostate cancer patients. J Cancer Educ. 2017;32(3):580–8. 10.1007/s13187-016-1025-2 27029194

[34] Kunneman M Engelhardt EG Ten Hove F, et al Deciding about (neo-) adjuvant rectal and breast cancer treatment: missed opportunities for shared decision making. Acta Oncol. 2016;55:134–9. 10.3109/0284186X.2015.1068447 26237738

[35] Charles C Gafni A Whelan T O’Brien MA Cultural influences on the physician-patient encounter: the case of shared treatment decision-making. Patient Educ Couns. 2006;63:262–7. 10.1016/j.pec.2006.06.018 17000073

[36] Couët N Desroches S Robitaille H, et al Assessments of the extent to which health-care providers involve patients in decision making: a systematic review of studies using the OPTION instrument. Health Expect. 2015;18:542–61. 10.1111/hex.12054 PMC5060794 23451939

[37] National Comprehensive Cancer Network. NCCN guidelines—breast cancer. 2018 Available from: https://www.nccn.org/professionals/physician_gls/default.aspx#breast.

[38] Gärtner FR Bomhof-Roordink H Smith IP Scholl I Stiggelbout AM Pieterse AH The quality of instruments to assess the process of shared decision making: a systematic review. PLoS One. 2018;13:e0191747. 10.1371/journal.pone.0191747 PMC5813932 29447193

